# The potential effect of metabolic alkalosis on insulin sensitivity in an adolescent with new‐onset type 1 diabetes

**DOI:** 10.1002/ccr3.3915

**Published:** 2021-05-24

**Authors:** William E. Novotny, Irma Fiordalisi, Cynthia P. Keel, Glenn D. Harris

**Affiliations:** ^1^ Brody School of Medicine East Carolina University Greenville NC USA; ^2^ Vidant Medical Center James and Connie Maynard Children’s Hospital Greenville NC USA

## Abstract

Metabolic alkalosis induced by ingestion of alkaline water may enhance insulin sensitivity in type 1 diabetes mellitus.

## Graphical abstract

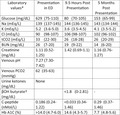

## 1 INTRODUCTION

Insulin deficiency in type 1 diabetes mellitus results in ketone body formation and eventual ketoacidosis. Metabolic acidosis is associated with insulin resistance. Consumption of alkaline drinks with associated metabolic alkalosis may prevent development of ketosis by enhancing residual insulin sensitivity.

Serum insulin concentrations associated with diabetic ketoacidosis (DKA) are lower than those associated with the hyperosmolar hyperglycemic state (HHS).^1^ As ketoacidosis develops, cellular sensitivity to insulin is impaired. It is unknown whether oral intake of metabolic alkali in the setting of hyperglycemia might enhance the effect of insulin and prevent development of ketosis.

## 2 METHODS: CASE REPORT

A case report of an adolescent who developed metabolic alkalosis and hyperglycemia without accompanying ketosis despite low insulin levels.

## 3 RESULTS

An athletic 60 kg African American adolescent boy presented with serum glucose 629 mg/dL, moderate dehydration and new onset diabetes mellitus. For the preceding two months, he had complained of fatigue and excessive thirst with polydipsia. Over the 2 weeks prior to presentation to the emergency department (ED), despite an excellent appetite, he lost approximately 9 kg and his basketball performance was impaired. Fluid intake over this period consisted of an average of 11.4 liters of fluid mainly TEN Alkaline Spring Water (pH 10.0) and some Gatorade (pH 2.9) every other day. On arrival to the ED, he complained of dizziness and vomited the first and only time during the span of his illness. His eyes were sunken; the Glascow Coma Score was 15. Initial vital signs: temperature 98⁰F/36.7⁰C, pulse 84/minute, blood pressure 143/89 mmHg, respiratory rate 15/min without Kussmaul breathing, oxygen saturation 99% on room air, weight 59.4 kg, and height 190.5 cm. Acanthosis nigricans was absent. Urine analysis revealed glucose ≥ 500 mg/dL, pH 7.0, and specific gravity 1.033. Initial effective osmolality was 313 mOsm/kg H_2_0 ^2^ and predicted sodium was 147 mEq/L. Additional initial laboratory results are listed in Table 1. One liter of 0.9% saline and 0.1 units/kg/hour of continuous regular insulin IV were administered.

**TABLE 1:** Laboratory values at presentation to emergency department and 9.5 hours and 5 months thereafter

Laboratory values^a^	Presentation in ED	9.5 h Postpresentation	5 mo Postpresentation
Glucose (mg/dL)	629 (75‐110)	80 (70‐105)	153 (65‐99)
Na (mEq/L)	139 (137‐145)	144 (136‐145)	143 (134‐144)
K (mEq/L)	5.2 (3.6‐5.0)	3.6 (3.5‐4.5)	4.1 (3.5‐5.2)
Cl (mEq/L)	90 (98‐107)	106 (98‐107)	102 (96‐102)
tCO2 (mEq/L)	33 (22‐30)	26 (18‐28)	26 (20‐29)
BUN (mg/dL)	26 (7‐20)	19 (9‐22)	14 (6‐20)
Creatinine (mg/dL)	1.11 (0.52‐1.25)	1.42 (0.69‐1.1)	1.16 (0.76‐1.27)
Venous pH	7.27 (7.30‐7.42)		
Venous PCO2 (mm Hg)	62 (35‐63)		
Urine ketones (mg/dL)	None		
βOH butyrate^b^ (mg/dL)		<1.8 (0‐2.81)	‐
C‐peptide (nMol/L)	0.186 (0.24‐1.22)	<0.033 (0.34‐1.46)	0.29 (0.37‐1.46)
Hb A1C (%)	>14.0 (4.7‐6.0)	14.6 (4.3‐5.7)	7.7 (4.8‐5.6)

^a^ Normal values shown in parentheses.

^b^ 3.43 (0.2‐2.8) at 16 h; <1.8 (0.2‐2.8) at 64 h.

Upon arrival to the pediatric intensive care unit (PICU), betahydroxybutyrate (βOH butyrate) was just above the reported normal reference range; the regular insulin infusion had been initiated six hours prior to obtaining this blood sample. Laboratory results obtained within 2 days of admission included negative islet cell antibody screen, and negative insulin antibodies, and GAD‐65 autoantibody <5 IU/mL; both antithyroid peroxidase and thyroglobulin antibody were hemolyzed; IgA 316 (53‐287 mg/dL), transglutaminase IgG 1 unit/mL (<6) and transglutaminase IgA 1 unit/mL (<4).

Dehydration was clinically assessed to be approximately 6%. Rehydration was planned over a 48‐hour period ^3^ with IV fluid containing 12.5% dextrose, NaCl 154 mEq/L and KPO4 20 mEq/L, KCl 10 mEq/L, KAc 10 mEq/L. Twenty‐four hours into the planned rehydration phase serum sodium was 145 mEq/L, potassium 4.6 mEq/L, chloride 105 mEq/L and total CO_2_ (tCO_2_) 27 mEq/L. Regular insulin infusion was initiated at 0.03 unit/kg/hour for approximately 10 hours before converting to long‐acting insulin. At discharge to home, insulin glargine 24 units at bedtime and insulin aspart 1 unit/10 grams carbohydrate were prescribed.

Five months after presentation and without further consumption of alkaline water, serum sodium was 143 mEq/L, tCO_2_ 26 mEq/L, BUN 14mg/dl, creatinine 1.16 mg/dL, Hb A1c 7.7%.

## 4 DISCUSSION

This report described an adolescent presenting in a nonketotic hyperglycemic state with an elevated tCO2. He had oral intake of large volumes of alkaline alkalinized water in the weeks prior to presentation. His physiologic condition was neither diabetic ketoacidosis (DKA) nor diabetic ketoalkalosis ^4^ because based on urine testing at presentation no ketone bodies were detected. The alkalosis may have been attributable to more than one factor ^5^: (a) excessive alkali intake, (b) dehydration with volume contraction alkalosis, (c) hypochloremia, (d) renal dysfunction, (e) compensation for respiratory acidosis, and (f) emesis. Follow‐up data did not suggest the presence of chronic respiratory acidosis. At time of presentation metabolic alkalosis was likely caused by a combination of alkali intake and dehydration with volume contraction.

At presentation, serum glucose measured 629 mg/dL with a simultaneously obtained insulin C‐peptide measuring only 0.186 (0.242‐1.225 nMol/L). C‐peptide insulin levels have been compared in patients with DKA and nonketotic hyperglycemic state; C‐peptide levels in DKA approximate 0.21 ± 0.03 nMol/L while in nonketotic hyperosmolal state levels are typically higher, in the range of 1.14 ± 0.10 nMol/L.^1^ In the setting of a low C‐peptide, most frequently associated with DKA, it is remarkable that ketosis was absent in urine at presentation to the outlying ED and absent in blood at presentation to PICU 9.5 hours into treatment course. Marked hyperglycemia (without ketosis) was likely present for a prolonged period as evidenced by the high HbA1C. In patients with type 1 diabetes, mellitus and ketosis altered degrees of insulin sensitivity may be present and may contribute to hyperglycemia.^6^ Experimentally induced chronic *metabolic acidosis* in healthy humans impairs cellular sensitivity to insulin ^7^, ^8^ and that slight variation of pH caused by excessive dietary acid load impairs insulin sensitivity.^7^ The initial venous blood gas reflected marked *respiratory acidosis* which was inconsistent with this child's alert, comfortable and interactive appearance, and more consistent with sampling error. A repeat venous blood gas was not performed to demonstrate the absence of respiratory acidosis. At follow‐up five months later, tCO2 was not elevated as might be expected if chronic hypercarbia had been present.

It is not known if metabolic *alkalosis* might enhance sensitivity to (low concentrations) of insulin in the setting of type 1 diabetes mellitus. By contrast, in normal, healthy adults increased levels of serum bicarbonate have been associated with indices reflecting higher insulin sensitivities.^9^ Insulin concentrations present in hyperglycemic hyperosmolar state appear to be adequate to sufficiently suppress lipolysis and ketogenesis but not sufficient to promote glucose utilization by insulin‐sensitive tissues.^10^ In one study of nondiabetic humans, preprandial bicarbonate supplementation before a high acid load meal did not appear to prevent postprandial glycemia or insulin response.^11^

Alkalinized water has been demonstrated to enhance hydration, improve acid–base balance as well as anaerobic exercise performance in high activity sport athletes.^12^, ^13^ Consumption of alkalinized water before and during physical activity by athletes has become a more frequent means of preventing and treating dehydration related to physical exertion. The adolescent reported here had been treating dehydration caused by both intense daily exercise but also dehydration related to severe persistent hyperglycemia. This case report serves to alert the clinician to the yet to be elucidated association of improved insulin sensitivity in athletes who consume alkali that are newly diagnosed with diabetes mellitus with severe hyperglycemia, very low insulin levels and no ketosis.

## 5 CONCLUSION

The very low C‐peptide concentration in the presence of a very high HbA1c% in this thin, athletic adolescent indicates type 1 diabetes mellitus. It is not known how exogenous base supplementation may affect insulin sensitivity in the diabetic patient. We speculate that the presence of a pre‐existing metabolic alkalosis may have enhanced insulin sensitivity and aided in the prevention of ketone body production.

## CONFLICT OF INTEREST

ICMJE's Uniform Disclosure Form for Potential Conflicts of Interest. To be forwarded. No conflict of interest existed for any author: William E. Novotny, Irma Fiordalisi, Cynthia P. Keel, Glenn D. Harris.

## AUTHOR CONTRIBUTIONS

William E. Novotny, Irma Fiordalisi, Cynthia P. Keel, Glenn D. Harris acquired data, drafted and revised the manuscript, provided final approval of the manuscript and agreed to be accountable for all aspects of the work.

## ETHICAL APPROVAL

The Institutional Review Board of East Carolina University Brody School of Medicine and Vidant Medical Center (the ethics committee) does not require approval of a deidentified report of a single patient. Nonetheless, signed consent for this case report was obtained from the parent of the child.

## CONSENT

No reproduction of material from other sources was included in this manuscript; no permission for reproduction was therefore obtained.

## Data Availability

The authors confirm that the data supporting the findings of this study are available within the article and/or its supplementary materials.
